# Quantitative real-time PCR detection and analysis of a lumpy skin disease outbreak in Inner Mongolia Autonomous Region, China

**DOI:** 10.3389/fvets.2022.936581

**Published:** 2022-07-26

**Authors:** Lin Li, Chuanxiang Qi, Jinming Li, Wenlong Nan, Ying Wang, Xing Chang, Tianying Chi, Mingxia Gong, Da Ha, Jide De, Lifeng Ma, Xiaodong Wu

**Affiliations:** ^1^China Animal Health and Epidemiology Center, Qingdao, China; ^2^MOE Joint International Research Laboratory of Animal Health and Food Safety, MOA Key Laboratory of Animal Bacteriology, College of Veterinary Medicine, Nanjing Agricultural University, Nanjing, China; ^3^Xilingol League Animal Disease Prevention and Control Center, Inner Mongolia, China; ^4^Inner Mongolia Animal Disease Prevention and Control Center, Inner Mongolia, China

**Keywords:** LSDV, quantitative real-time PCR (qPCR), China, outbreak, Inner Mongolia Autonomous Region, 2020

## Abstract

Lumpy skin disease (LSD) is a severe disease of bovine characterized by nodules on the skin, mucous membranes, and profuse nasal discharge which causes severe economic losses. In October 2020, an LSD outbreak case was found in Inner Mongolia Autonomous Region, China. A total of 1,206 cattle were sold from the same imported animal quarantine field to 36 farms after the quarantine period finished, and over 30 farmers reported symptoms such as skin scabs found in newly arrived cattle shortly after that. A large-scale LSD outbreak investigation was launched after laboratory diagnosis confirmed LSD. The clinical samples of 1,206 cattle from 36 farms, including 1,206 whole blood, 1,206 oral and nose swabs, and 355 scabs, were collected for the qRT-PCR test. The result showed that 51 whole blood samples (4.23%), 580 swab samples (48.09%), and 350 skin scabs (98.59%) were lumpy skin disease virus (LSDV) positive, 33 of 36 farms were affected. This study aims to provide a basis for LSD epidemiological traceability, movement control, and measures for prevention and control.

## Introduction

Lumpy skin disease (LSD) is a severe disease of bovine characterized by multifocal cutaneous nodules which were caused by the lumpy skin disease virus (LSDV). LSDV is classified into the genus *Capripoxvirus* of the family *Poxviridae*. As a large double-stranded DNA virus, LSDV encodes 156 putative viral genes with a genome of 151 kb (1). Unlike other *Capripoxvirus* which infect sheep and goats, LSDV is highly host-specific and mainly infects cattle and buffalo under natural conditions. The severity of clinical signs of LSD varies from subclinical to fatal depending on the virulence of the strains and the host susceptibility.

Lumpy skin disease SD outbreaks caused severe economic losses due to decreased milk production and weight gain, abortions, and reduced quality of material (2, 3) resulting in a great impact on both the national and world livestock industry. LSDV could be transmitted by bloodsucking vectors such as mosquitoes, midges, and stable flies (4), circulating in the affected areas, which poses certain difficulties for LSDV prevention and control. In 1929, LSD was first reported in Zambia, and in the next few decades, it spread throughout southern Africa and north to Sudan, and then to the Middle East and further spread to Europe and western Asia. After that, LSDV spread rapidly to Southeast Asia and to Kazakhstan (5, 6). In August 2019, LSD was first confirmed in China near to Kazakhstan border. Since 2020, several outbreaks were reported in eastern and southern China sporadically (7).

Although the incubation period under field conditions of LSDV usually lasts for 1–4 weeks in natural outbreaks (6) and then LSDV can be detected in the lesions of diseased animals. Once the cattle are infected with LSDV, the nodules on the mucous membranes of the eyes, nose, mouth, rectum, udder, and genitalia quickly ulcerate, and by then, all secretions, ocular and nasal discharge, and saliva contain LSDV. LSDV could be detected in skin nodules, normal skin, lymph nodes, liver, kidneys, skeletal muscle, saliva, and semen of naturally and experimentally animals (8, 9).

Rapid and precise diagnosis and confirmation of suspected cases are important for the successful control and eradication of LSD in endemic and especially non-endemic countries. LSD laboratory testing involves conventional gel-based PCR (3), RT-PCR (10–12), and HRM-based methods (13) that have been developed and validated for rapid detection of the LSDV genome. Among these methods, RT-PCR as a rapid, reliable, sensitive, and specific method has been widely used in recent years.

In October 2020, some suspected cases of LSD were reported in Xilingol League, Inner Mongolia Autonomous Region, China. A total of 36 farms with 1,206 cattle were involved. Clinical samples including oral and nose swabs, whole blood, and scabs were collected two weeks later, and the molecular diagnosis was carried out immediately in a BSL-2 laboratory at China Animal Health and Epidemiology Center (CAHEC). The purpose of this study is to describe the results of using RT-PCR to detect LSDV in infected cattle specimens in order to help the surveillance of LSD.

## Materials and methods

### Sample collection

**Figure 1:**
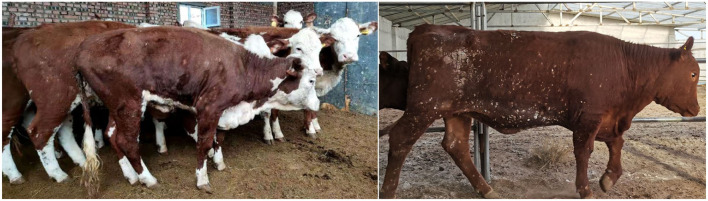
Cattle affected with the lumpy skin disease virus present nodules.

Clinical samples including anticoagulant blood, oral and nasal swabs, and scabby samples were collected by local veterinarians from 1,206 cattle on 36 farms. For those cattle with scabs (Figure 1), oral and nasal swabs (ON), whole blood (WB), and scab samples were collected, for others, only oral and nasal swabs and whole blood were taken for diagnosis. The blood samples were stored in anticoagulant blood vessels and other samples were kept in sterile centrifuge tubes with clear labels. A total of 1,206 blood samples, 1,206 swab samples, and 355 scab samples were collected. All samples were transported under low-temperature conditions (ice bag) for quality maintenance.

### Sample preparation and viral DNA extraction

The samples (oral and nasal swabs, whole blood, and scabs) were thawed at room temperature. Oral and nasal swabs samples in phosphate buffer solution (PBS, pH 7.4) were infiltrated sufficiently before nucleic acids were extracted. Scab samples were cut into small pieces weighing about 200 mg and homogenated in 1,000 μl PBS by JXCL-3K Cryomill (Jingxin, Shanghai, China) followed by a centrifuge (3 min, 5,000 rpm) for supernatant. Whole blood samples were directly used in the next step of nucleic acid extraction. 200 μl of each preliminary prepared sample was used for further nucleic acid extraction. Total DNA/RNA was automatically extracted by Tianlong NP968-C Nucleic Acid Extractor with the GeneRotex96 program (TianLong medtl, Xi'an, China). Finally, the total DNA/RNA was dissolved in 80 μl elution buffer and stored at −20°C for further use. All samples were processed in a BLS-2 laboratory.

### Real-time PCR (qPCR)

A TaqMan-based qRT-PCR analysis was used with a pair of specific primers and a probe (Table 1) target to putative virion core gene, LSDV101, which were synthesized by Sangon Biotech Co., Ltd. (Shanghai). RT-PCR amplifications were carried out in a 25 μl reaction system containing 5 μl extracted sample nucleic acid or template controls and 20 μl of the prepared master mix. PCR master mix consists of 12.5 μl 2×Taq MasterMix (Vazyme, Nanjing, China), 1 μl of each primer,.5 μl probe, and 5 μl RNase-free ddH_2_O. The thermocycling conditions for the PCR were 50°C for 2 min, 95°C for 5 min, and then 40 cycles of amplification (95°C for 15 s, 58 °C for 15 s), FAM (5-carboxyfluorescein) signal was collected at 58°C at each cycle for Quantitative analysis. The reaction was run on a QuantStudio 5 PCR instrument (Thermofisher, USA), and a CT value <40 was judged as a positive sample.

**Table 1:** Primers and probe used in this study.

**Name**	**Primers (5**′**-3**′**)**	**Size (bp)**
LSDV-F	TGAATTAGTGTTGTTTCTTC	59bp
LSDV-R	GGGAATCCTCAAGATAGTTCG	
LSDV-P	FAM-TGCCGCAAAATGTCGA-MGB	

### Statistical analysis

All statistical analyses used a two-tailed Student's *t*-test and graphics were performed by R programming (14).

## Results

### Clinical

According to the investigation, all the cattle of the affected farms were related to the same place in east China. A total of 36 farms with 1,206 cattle were involved, and clinical symptoms including nodes, swollen joints, and blindness were observed in 31 farms since October 2020. Clinical samples including 1,206 oral and nose swabs, 1,206 whole blood, and 355 scabs were collected 2 weeks later.

### Real-time PCR testing

A total of 2,767 samples were tested by RT-PCR for the presence of LSDV from 1,206 cattle, including 1,206 blood samples, 1,206 swab samples, and 355 scab samples. As shown in Table 2, 350 of 355 scab samples (98.59%) and 580 out of 1,206 swab samples (48.09%) were detected as LSDV positive, and the positive sample number in blood was 51 out of 1,206 (4.23%).

**Table 2:** Summary of qPCR results by clinic sign.

**Clinical** **sign**	**Scab pos**.	**Swab pos**.	**Whole blood pos**.
With Scab (*n* = 355, 29.44%)	350 (98.59%)	228 (64.23%)	19 (5.35%)
No Scab (*n* = 851, 70.56%)	N/A	352 (41.36%)	32 (3.76%)
Total (*n* = 1206)	350 (29.02%)	580 (48.09%)	51 (4.23%)

**Figure 2:**
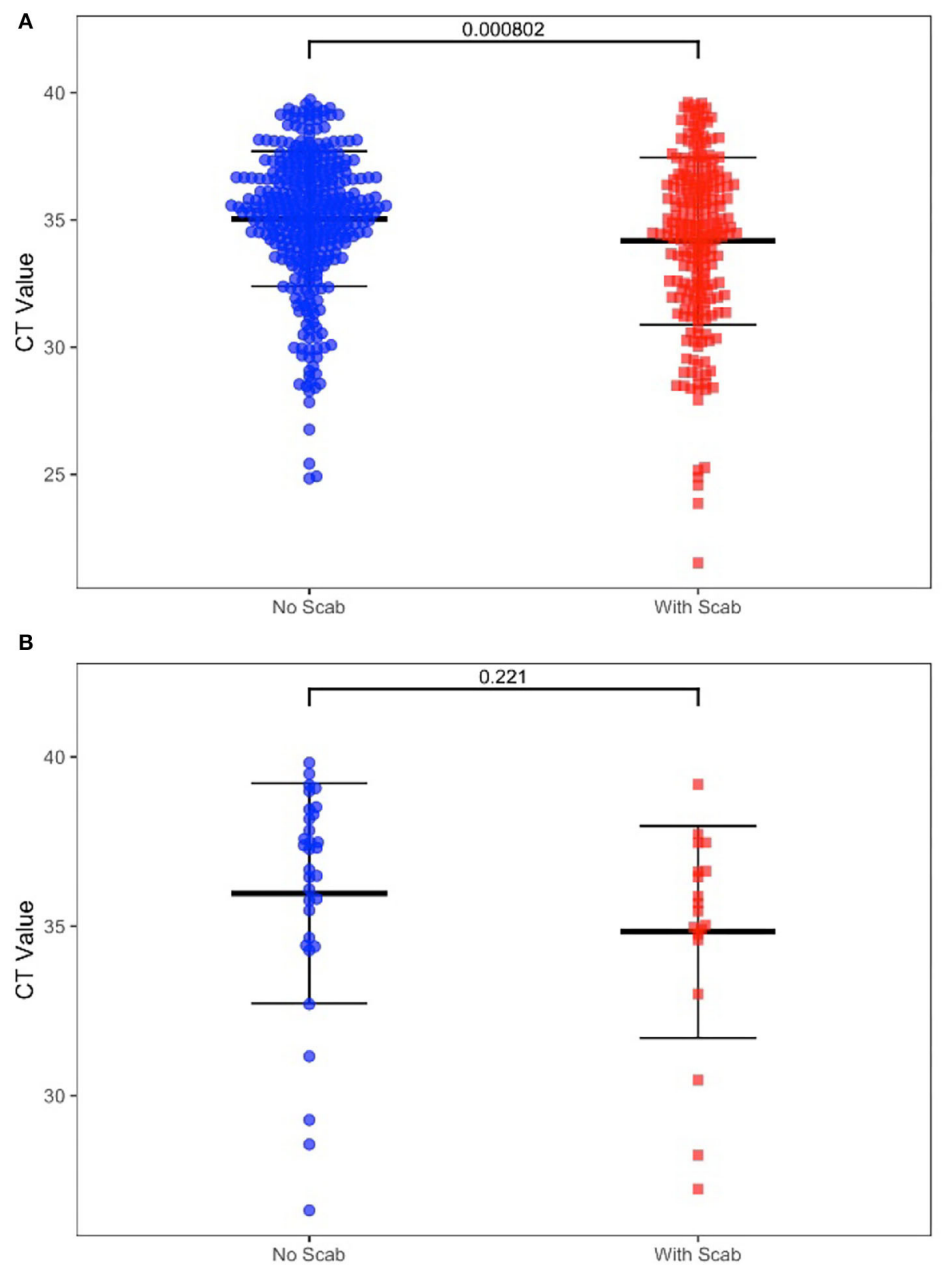
**(A)** Diagnostic results of positive swab samples by clinic sign. **(B)** Diagnostic results of positive whole blood samples by clinic sign.

We also determined the positive rate of the samples according to whether the cattle had clinical symptoms in Table 2. Among 1,206 cattle, 355 (29.44%) displayed positive symptoms with scabs, in this group, 350 scab samples (98.59%), 228 swab samples (64.23%), and 19 whole blood samples (5.35%) were confirmed as LSDV positive. The remaining cattle (70.56%) showed no clinical signs, in this group, 352 swab samples (41.36%), and 32 whole blood samples (3.76%) were confirmed as LSDV positive. For those positive swab samples, the CT value was significantly different (*P*-value = 0.0008) between the two groups. However, CT values in positive blood samples show no significant difference (*P* = 0.221) between groups with or without clinical signs when compared (Figures 2A,B).

**Figure 3:**
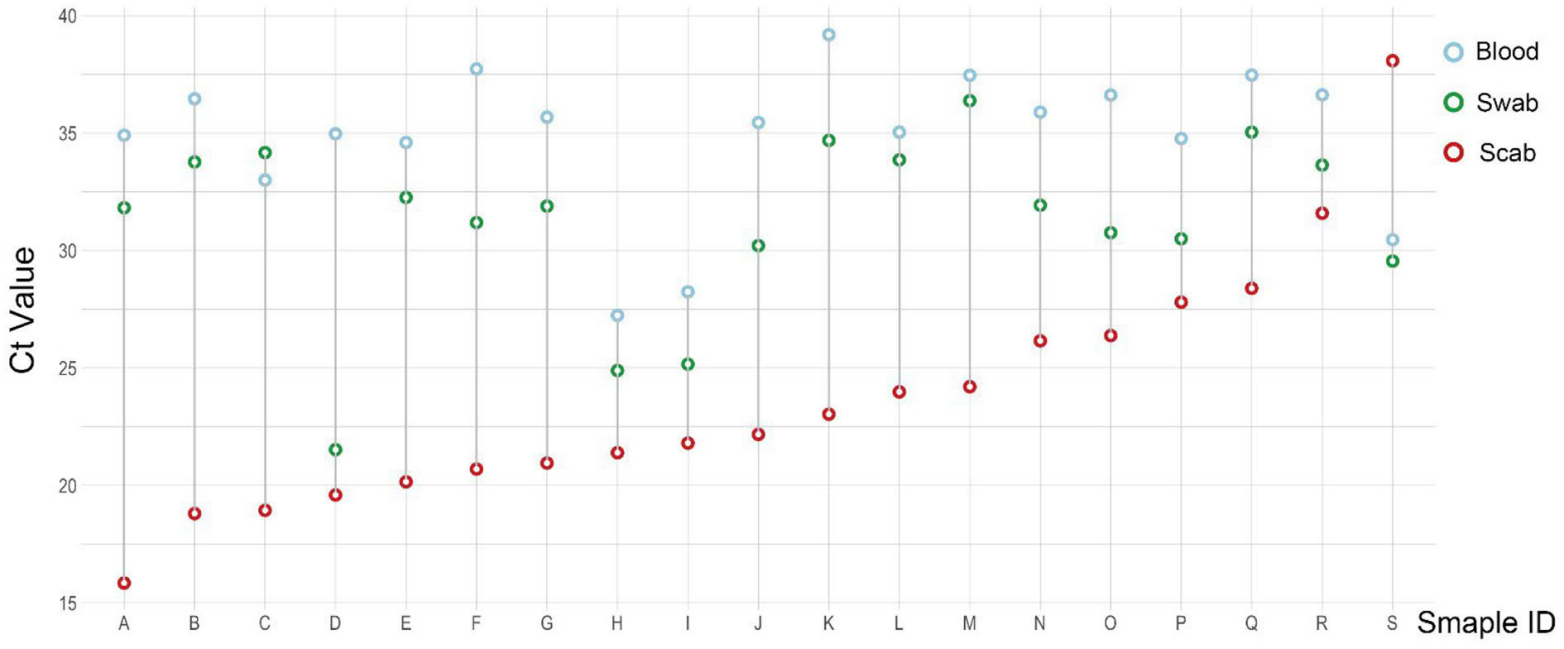
Ct value distribution in 19 scab-swab-blood positive samples.

As the result of this test, a total of 19 cattle were confirmed LSDV positive for all three types of samples (Figure 3). For each cattle with a few exceptions, the CT value of the scab sample (red circle) was the lowest, and then comes with the swab sample (green circle), the CT value of the blood sample (blue circle) was the highest when compared with the other two types of samples.

**Figure 4:**
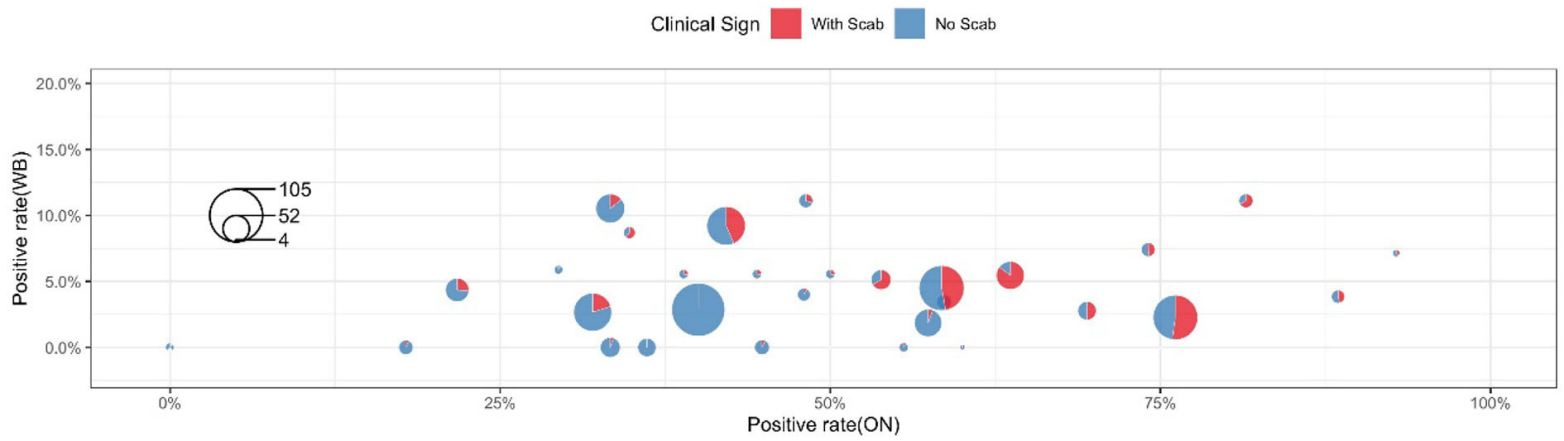
Sample testing results of each individual farm.

Table 3 shows the clinical diagnosis of LSD in cattle by village and farm. Most farms can find clinical symptoms and provide warning references for prevention and control, but there are cases of asymptomatic subclinical infection in a few farms, which should not be ignored. Figure 4 shows a summary of the sample test results of 36 farms. In this figure, each circle represents a farm and the size was herd number dependent, each circle consists of two parts: the blue part represents the proportion without LSD clinical signs while the rest red part represents LSD symptoms positive. The X-axis shows the positive rate of nasal swabs on the farm and the Y-axis represents the positive rate of whole blood samples on the farm. The figure showed that the positive rate of whole blood samples was much lower than that of swab samples. In addition, cattle with scabs had a higher percentage of nasal swabs, and whole blood was identified as positive by RT-PCR.

**Table 3:** Clinical diagnosis of LSD in cattle by village and farm.

**Farm ID**	**Herd Size**	**Cattle with clinical symptom**	**Scab sample pos**.	**Blood sample pos**.	**Oral & nose sample pos**.	**Disease incidence**	**Scab sample pos. (Ratio)**	**Blood sample pos**. **(Ratio)**	**Oral & nose sample pos. (Ratio)**
Farm01	46	12	8	2	10	26.09%	66.67%	4.35%	21.74%
Farm02	88	46	45	2	67	52.27%	97.83%	2.27%	76.14%
Farm03	89	42	42	4	52	47.19%	100.00%	4.49%	58.43%
Farm04	36	18	18	1	25	50.00%	100.00%	2.78%	69.44%
Farm05	76	33	33	7	32	43.42%	100.00%	9.21%	42.11%
Farm06	26	12	12	1	23	46.15%	100.00%	3.85%	88.46%
Farm07	23	14	14	2	8	60.87%	100.00%	8.70%	34.78%
Farm08	75	15	15	2	24	20.00%	100.00%	2.67%	32.00%
Farm09	18	5	5	1	8	27.78%	100.00%	5.56%	44.44%
Farm10	18	5	5	1	9	27.78%	100.00%	5.56%	50.00%
Farm11	27	8	8	3	13	29.63%	100.00%	11.11%	48.15%
Farm12	27	18	18	3	22	66.67%	100.00%	11.11%	81.48%
Farm13	18	2	2	0	10	11.11%	100.00%	0.00%	55.56%
Farm14	27	13	13	2	20	48.15%	100.00%	7.41%	74.07%
Farm15	17	1	1	1	5	5.88%	100.00%	5.88%	29.41%
Farm16	18	5	5	1	7	27.78%	100.00%	5.56%	38.89%
Farm17	55	47	47	3	35	85.45%	100.00%	5.45%	63.64%
Farm18	39	26	26	2	21	66.67%	100.00%	5.13%	53.85%
Farm19	14	5	5	1	13	35.71%	100.00%	7.14%	92.86%
Farm20	4	1	1	0	2	25.00%	100.00%	0.00%	50.00%
Farm21	10	3	3	0	6	30.00%	100.00%	0.00%	60.00%
Farm22	4	1	1	0	3	25.00%	100.00%	0.00%	75.00%
Farm23	4	2	2	0	0	50.00%	100.00%	0.00%	0.00%
Farm24	57	8	8	6	19	14.04%	100.00%	10.53%	33.33%
Farm25	25	2	2	1	12	8.00%	100.00%	4.00%	48.00%
Farm26	29	1	1	1	17	3.45%	100.00%	3.45%	58.62%
Farm27	39	2	2	0	13	5.13%	100.00%	0.00%	33.33%
Farm28	15	1	1	0	0	6.67%	100.00%	0.00%	0.00%
Farm29	28	2	2	0	5	7.14%	100.00%	0.00%	17.86%
Farm30	54	3	3	1	31	5.56%	100.00%	1.85%	57.41%
Farm31	29	2	2	0	13	6.90%	100.00%	0.00%	44.83%
Farm32	7	0	0	0	0	0.00%	/	0.00%	0.00%
Farm33	36	0	0	0	13	0.00%	/	0.00%	36.11%
Farm34	5	0	0	0	0	0.00%	/	0.00%	0.00%
Farm35	105	0	0	3	42	0.00%	/	2.86%	40.00%
Farm36	18	0	0	0	0	0.00%	/	0.00%	0.00%

## Discussion

Lumpy skin disease, a viral transboundary animal disease, is one of the bovine notifiable diseases by the World Organization for Animal Health (OIE, https://www.oie.int/en/disease/lumpy-skin-disease/) which causes a serious socio-economic impact. In 2013, LSD spread to Europe and was subsequently disseminated in 11 European countries, including Turkey, Greece, and Russia (15–17). From 2019 on, outbreaks of LSD have been reported by central Asian and neighboring countries, such as India, Bangladesh, Nepal, and China (7, 18). China first identified LSD in northwest Xinjiang, in 2019, and multiple provinces reported LSD outbreaks shortly after that (7, 18, 19). In order to prevent LSDV from further spreading, great efforts for disease prevention and control must be taken: raising biosafety awareness, increasing public knowledge of LSDV, reducing the density of LSDV vectors, strengthening the regulation of illegal animal movements, implementing massive vaccination campaigns in affected areas with GPTV vaccine (AV41 Strain). However, the vaccination process varies between provinces and no effective measures to control the blood-sucking insect vectors, the risk of LSDV infection is high in outdoor-raised, unvaccinated or immunocompromised cattle.

In this study, test results of samples collected in parallel from 350 clinically symptomatic cattle showed that the positive detection rate of scab samples was 98.59%, the positive detection rate of oral and nasal swabs was 64.86%, and the positive detection rate of whole blood samples was 5.43%. The CT values of cattle with positive skin scabs, whole blood, and oral and nasal swab samples were all found to be significantly different. In 19 scab-swab-blood positive samples, the CT value of skin scabs was the lowest, indicating that this sample was the most sensitive. The results show that cattle with clinical symptoms of pimples and scabs on the skin can be used as a reference for the preliminary judgment of farmers and grass-roots veterinarians. An interesting fact is that we detected 5 scabs negative but swab positive samples. The study by Babiuk et al. has shown that a skin nodule was negative by RT-PCR and virus isolation indicating that skin lesions must be confirmed to be caused by LSD (20), so here we speculate that scabs may be caused by other skin lesions, remnants of rehabilitation, or even trauma while the swab positive may be caused by environmental contamination.

Among the parallel three samples collected, whole blood samples show disadvantages including time-consuming, short validity window period, and cattle injury brought from invasive sampling. Studies have shown that the presence of LSDV in the blood is short-lived between 6–15 days, viremia peaked at 9 days and continued to decline rapidly post-infection, while the virus is present in scabs after the presence of LSDV has been cleared from mucosal secretions and could be detected in swabs for a longer duration between 12–21 days (20). Our results also showed the lowest detection rate of LSDV in whole blood samples suggests that whole blood samples may not be suitable for clinical diagnosis of LSDV. Another sample is a scab, scab samples were easy to get for clinical examination, and in this test, scab samples were also the most sensitive clinical samples as they showed the highest detection rate close to 100%, and the median positive detection rate in scabs was also 100%, such a reliable diagnosis result emphasizes scab samples play a dominant role in LSDV positive discrimination and can be used as the decisive diagnostic basis. However, an indisputable fact is that once the symptoms of scabbing found in the herd usually indicate that LSDV infection in the herd for a period of time, thus scab symptoms be an important basis for farmers to judge the health status of cattle in the early stage. Oral and nasal swab samples can be obtained easily by a simple operation in the clinic, the whole collection process is not invasive to cattle thus the harm to animals can be almost ignored. In this study, our data also showed reliable discrimination results in swab samples. Although more precise LSDV infection rate results can be achieved by combining the oral and nasal swab samples and the whole blood sample, limited conditions usually impaired sample collection. In view of this, we prefer to recommend oral and nasal swabs for the clinical diagnosis of large-scale LSDV under limited conditions.

For 851 cattle without clinical symptoms, the results of the two samples showed that the positive detection rate of oral and nasal swabs was 41.36% and whole blood samples were 3.76%. The proportion of clinical symptoms varies in each factory as some farms with a low proportion of crusted cattle were found to have a high swab positive close to 40% (Farm 33 and Farm 35, Figure 4). These data suggested that a considerable number of cattle show subclinical infection. These apparently clinical healthy cattle may be in the early stage of infection and thus have not yet shown clinical symptoms but virus shed at mucosal sites *in vivo*. There is also a small chance that those cows with positive nasal swabs are actually uninfected cows due to the high possibility of environmental pollution. Considering these sub-clinically infected herds still have the risk of transmitting LSDV, it is necessary to strictly restrict cattle transportation and take preventive measures to control the further spread of LSDV.

Real-time PCR is a rapid, sensitive, and specific method for confirming capripox viruses including LSD (12). In this study, we found over one-third of all samples from tested animals were positive for the presence of LSDV viral DNA, this result is especially obvious in skin scab samples as they were consistently diagnosed positive, and skin scab samples were demonstrated by lower average CT values in PCR testing in this study proved that a greater average viral concentration exists in skin scab than other samples. We also noticed that nearly half of swab samples were detected as LSDV positive which shows a much lower LSDV positive ratio when compared with that in scab samples. However, a low detection rate does not hinder the necessary position of swab samples in the identification of LSDV as they can be collected at a very early stage for LSDV epidemic monitoring. For blood samples, we found that only 4.23% of samples were LSDV positive means that LSDV DNA was less likely to be detected in cattle' s blood, this result was consistent with the findings of studies that revealed LSD viremia to be a very short-lived-blood samples were positive for PCR for 4–11 days after infection, whereas virus was discovered in skin lesions for up to 92 days (3, 21). In our study, a 2-week interval existed between the cattle arriving at the ranch and the investigation started, this long sampling interval is likely to result in exceeding the optimal detection period for viremia, which may be the main reason for the low detection rate of the whole blood samples.

In summary, we analyzed RT-PCR results from one LSD outbreak case in Inner Mongolia Autonomous Region, China. We recommend that when collecting LSD epidemic samples, emphasis should be focused on scab samples and oral and nasal swab samples. More attention should be attached to the epidemiological source and mobile control of the same breed of cattle with negative clinical results.

## Data availability statement

The raw data supporting the conclusions of this article will be made available by the authors, without undue reservation.

## Author contributions

LL, CQ, JL, and XW designed the study, analysed data, and drafted the manuscript. CQ, WN, YW, XC, TC, and MG performed the experiments with help from DH and JD. LL, CQ, and JL developed the concept and interpreted the data. JL, LM, and XW supervised the entire project. All authors read and approved the final manuscript.

## Funding

This work was supported by the National Key Research and Development Program of China (2017YFD0501800).

## Conflict of interest

The authors declare that the research was conducted in the absence of any commercial or financial relationships that could be construed as a potential conflict of interest.

## Publisher's note

All claims expressed in this article are solely those of the authors and do not necessarily represent those of their affiliated organizations, or those of the publisher, the editors and the reviewers. Any product that may be evaluated in this article, or claim that may be made by its manufacturer, is not guaranteed or endorsed by the publisher.
